# Combination therapeutics in complex diseases

**DOI:** 10.1111/jcmm.12930

**Published:** 2016-09-07

**Authors:** Bing He, Cheng Lu, Guang Zheng, Xiaojuan He, Maolin Wang, Gao Chen, Ge Zhang, Aiping Lu

**Affiliations:** ^1^ Institute for Advancing Translational Medicine in Bone & Joint DiseasesSchool of Chinese MedicineHong Kong Baptist UniversityHong KongChina; ^2^ Institute of Integrated Bioinformedicine & Translational ScienceHKBU Shenzhen Research Institute and Continuing EducationShenzhenChina; ^3^ Institute of Basic Research in Clinical MedicineChina Academy of Chinese Medical SciencesBeijingChina

**Keywords:** combination therapeutics, complex diseases, molecular network

## Abstract

The biological redundancies in molecular networks of complex diseases limit the efficacy of many single drug therapies. Combination therapeutics, as a common therapeutic method, involve pharmacological intervention using several drugs that interact with multiple targets in the molecular networks of diseases and may achieve better efficacy and/or less toxicity than monotherapy in practice. The development of combination therapeutics is complicated by several critical issues, including identifying multiple targets, targeting strategies and the drug combination. This review summarizes the current achievements in combination therapeutics, with a particular emphasis on the efforts to develop combination therapeutics for complex diseases.

## Introduction

Living systems in the human body are interconnected networks of molecular components 1. Multiple complex molecular networks are involved in the mechanisms of many common diseases, especially in complex diseases (*e.g*., arthritis, diabetes, heart disease, cancer and asthma) 2, 3, 4, 5, 6. The complexity of these diseases limits the efficacy of commonly used single molecular drug therapeutics, which has led to a critical situation in new drug discovery in recent years 7. The strategy of combination therapeutics has been revisited with the hope of addressing this issue 8.

**Figure 1:**
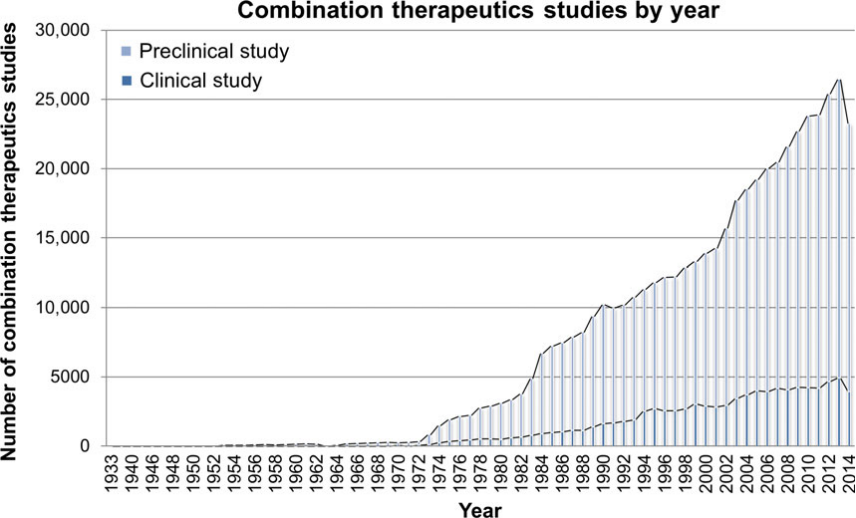
Statistics of combination therapeutic studies by year. The data were obtained from the PubMed database.

The number of studies investigating combination therapeutics is increasing annually (Fig. 1). Moreover, some drug combinations have entered clinical trials for the treatment of complex diseases (Table 1). This review will summarize the current achievements in combination therapeutics in complex diseases, with a particular focus on the computational strategy for drug combination.

**Table 1:** Recent clinical trials of combination therapeutics used for the treatment of complex diseases. We obtained data from articles published in *Lancet* and *The New England Journal of Medicine* in the previous 3 years

Disease	Combination therapeutics
Hypertension	Nebivolol + valsartan 33
Pyelonephritis	Ceftolozane + tazobactam 98
Membranous nephropathy	Prednisolone + chlorambucil 99
Diabetes	Dulaglutide + lispro 100
Rheumatoid arthritis	Adalimumab + methotrexate 101; Tofacitinib + methotrexate 102
Myeloma	Lenalidomide + dexamethasone+ carfilzomib 103; Lenalidomide + dexamethasone 104, 105
Colorectal cancer	Capecitabine + bevacizumab 106; FOLFOXIRI (fluorouracil, leucovorin, oxaliplatin, and irinotecan)+ bevacizumab 107
Pancreatic cancer	Gemcitabine + nanoparticle albumin‐bound (nab)‐paclitaxel (Abraxane) 108, 109, 110
Chronic lymphocytic leukaemia	Obinutuzumab + chlorambucil 111
B‐cell lymphoma	Rituximab + cyclophosphamide + doxorubicin + vincristine + prednisolone 112
Breast cancer	Pertuzumab + trastuzumab + docetaxel 113; Paclitaxel + trastuzumab 114
Melanoma	Trametinib + dabrafenib 115, 116; Nivolumab + Ipilimumab 117, 118; Dabrafenib + trametinib 119

## Combination therapeutics and drugs with multiple targets

Combination therapeutics are concerted pharmacological interventions consisting of several drugs that interact with multiple targets. The combination of drugs may be antagonistic, additive or synergistic if the combined effect is less than, equal to or greater than the sum of the individual drugs, respectively 9. Synergistic drug combinations have numerous advantages over monotherapy, including increased efficacy, decreased dosage with equal efficacy, reduced side effects and reduced drug resistance 10. These advantages are mainly due to the targeting of multiple molecular networks in the human body. Complex molecular networks are involved in various biological functions of the human body. For example, bone homeostasis, the process of creating new bone and removing old bone 11, has been found to be regulated by the WNT protein signalling pathway 12, the Notch signalling pathway 13, the RANK signalling pathway 14 and the bone morphogenetic protein (BMP) signalling pathway 15. Moreover, the molecular networks in the human body are also likely to be modulated by redundant pathways, which can compensate for one another if either one is inhibited. For example, BMP induces transcriptional activation mediated by SMAD1, SMAD5 and SMAD8, which functionally compensate for each other in various biological processes such as bone formation 16, trigeminal ganglia subtype specification 17 and stem cell differentiation 18. Such pathways are therefore optimally inhibited by multiple drugs that block all SMAD1/5/8 pathways, which ensures that the other pathways do not partially or completely compensate for the inhibition 16. Blocking the upstream signal (for example, the BMP receptor in this case) would not be appropriate because although it could inhibit all SMAD1/5/8 pathways, it would also block other downstream BMP signalling pathways, which could potentially lead to undesirable effects.

In addition to combination therapeutics, one drug might also target multiple functions 19. A drug that is designed to target multiple processes is an adjusted combination therapeutic in single drug form 20. Multi‐target drugs might overcome molecular heterogeneity within or between patients diagnosed with complex diseases, such as non‐small‐cell lung cancer 21, gastrointestinal stromal tumours 22 and pulmonary fibrosis 23, and would therefore have a better chance of successful clinical development, especially when biomarkers for patient selection are uncertain. However, such a multi‐target drug might not be ideal for reasonable therapeutic strategies based on specific molecular profiles of diseases. Because these drugs are not well ‘designed’ to interact with a particular set of targets, they may not achieve optimal inhibition of the specific individual targets in a particular disease 24. For example, during kinase inhibition, screening to detect the inhibitory activities of particular kinases of interest resulted in the identification of multi‐targeted kinase inhibitors. However, additional kinases with structural similarities will be inhibited, potentially leading to additional toxicity because cumulative target and off‐target inhibition has a broader, and perhaps less predictable, effect on cellular functions 24, 25. For example, Sorafenib, a multi‐target tyrosine kinase inhibitor with potent anti‐angiogenic activity that is used to treat solid tumours, induces irreversible pancreatic atrophy in patients after long‐term administration 26.

In contrast, the combination of more specific targeted drugs may be more suitable for therapeutics tailored to individual patients based on the specific molecular disease profiles 27. Physicians might adjust the concentrations of drugs within a combination therapeutic to enhance targeted inhibition and to optimize synergy between drugs 28. The toxicity of the combination might also be more predictable due to limited off‐target effects 29.

## Drug selection for combination therapeutics

Currently, most of the drugs in combination therapeutics are chemosynthetic molecules. For example, Ser‐Ap‐Es^®^, which has been used for the treatment of hypertension since the 1950s, is a triple‐chemical‐drug combination that includes reserpine, apresoline and hydrochlorothiazide. Hypertension is a complex cardiovascular disease, and approximately 30% of the adult population in the United States suffers from this condition 30. Law *et al*. have suggested that low‐dose combinations from the five major classes of antihypertensive drugs (diuretics, beta‐blockers, angiotensin‐receptor blockers, calcium‐channel blockers and angiotensin‐converting enzyme inhibitors) are more effective for reducing blood pressure and associated with fewer side effects than monotherapy 31. Taylor and Ragbir evaluated some triple‐drug combinations and showed that they were well‐tolerated with a low incidence of side effects 32. Recently, a fixed‐dose combination of a vasodilating β‐blocker (nebivolol) and an angiotensin II receptor blocker (valsartan) was shown to be an effective and well‐tolerated treatment option for patients with hypertension in an 8‐week, phase 3, multi‐centre, randomized, double‐blind, placebo‐controlled, parallel‐group trial 33.

Bioengineering products are becoming another important source of drugs for combination therapeutics. Monoclonal antibodies are the most common form of this type of drug 34. For example, trastuzumab, a monoclonal antibody that interferes with the HER2 receptor, has been used in combination with cisplatin to inhibit the progression of gastric cancer 35. Cisplatin induces DNA damage and apoptosis, which may be attenuated by DNA repair systems 36. Trastuzumab suppresses the DNA repair pathway and the PI3K‐AKT pathway 37 to increase cellular apoptosis 38. The combination of ramucirumab, a human monoclonal antibody against VEGFR2, and paclitaxel significantly increased the overall survival of gastric cancer patients in a randomized, placebo‐controlled, double‐blind, phase 3 trial in 27 countries in North and South America, Europe, Asia and Australia 39.

Herbs have been widely used in eastern Asia for thousands of years. Herbal compounds would be good candidates for combination therapeutics. There are already several successful cases examining this theory. Paclitaxel, a drug developed from a compound isolated from the bark of the pacific yew tree, *Taxus brevifolia*, is now widely used in combination therapeutics for cancer treatment. The combination of paclitaxel and bortezomib, a proteasome inhibitor that modulates apoptosis and the cell cycle by disrupting protein degradation, improves overall survival of non‐small cell lung cancer patients 40. Total glucosides of peony, active compounds extracted from the roots of the herb *Paeonia lactiflora* Pall, reduce hepatotoxicity in combination with leflunomide and methotrexate in patients with active rheumatoid arthritis 41. In China, the herb *Artemisia annua* is used to treat fever and malaria. In 1971, artemisinin was isolated from the leafy portions of the *A. annua* plant and found to be effective for the treatment of malaria 42. Artemisinin‐combination therapy is now recommended as a first‐line treatment for falciparum malaria worldwide, and fixed‐dose combinations are preferred by the WHO. In addition, some combinations of herbal compounds are in preclinical phases. Deng *et al*. have indicated that the combination of salvianolic acid B and ginsenoside Rg1, which are derived from roots of *Salvia miltiorrhiza* and *Panax notoginseng*, respectively, improve the viability of cardiac myocytes in rats with ischaemia/reperfusion injury 43. Zhang *et al*. have found that tetramethylpyrazine, a compound isolated from *Ligusticum wallichii Franchat,* ameliorates oxidative organ injury associated with methotrexate treatment in patients with rheumatoid arthritis 44. These examples illustrate the possibility of utilizing herbal compounds in combination therapeutics. Therefore, herb might be a rich resource for the discovery of combination therapeutics.

## Drug combinations are much more than synergistic

**Figure 2:**
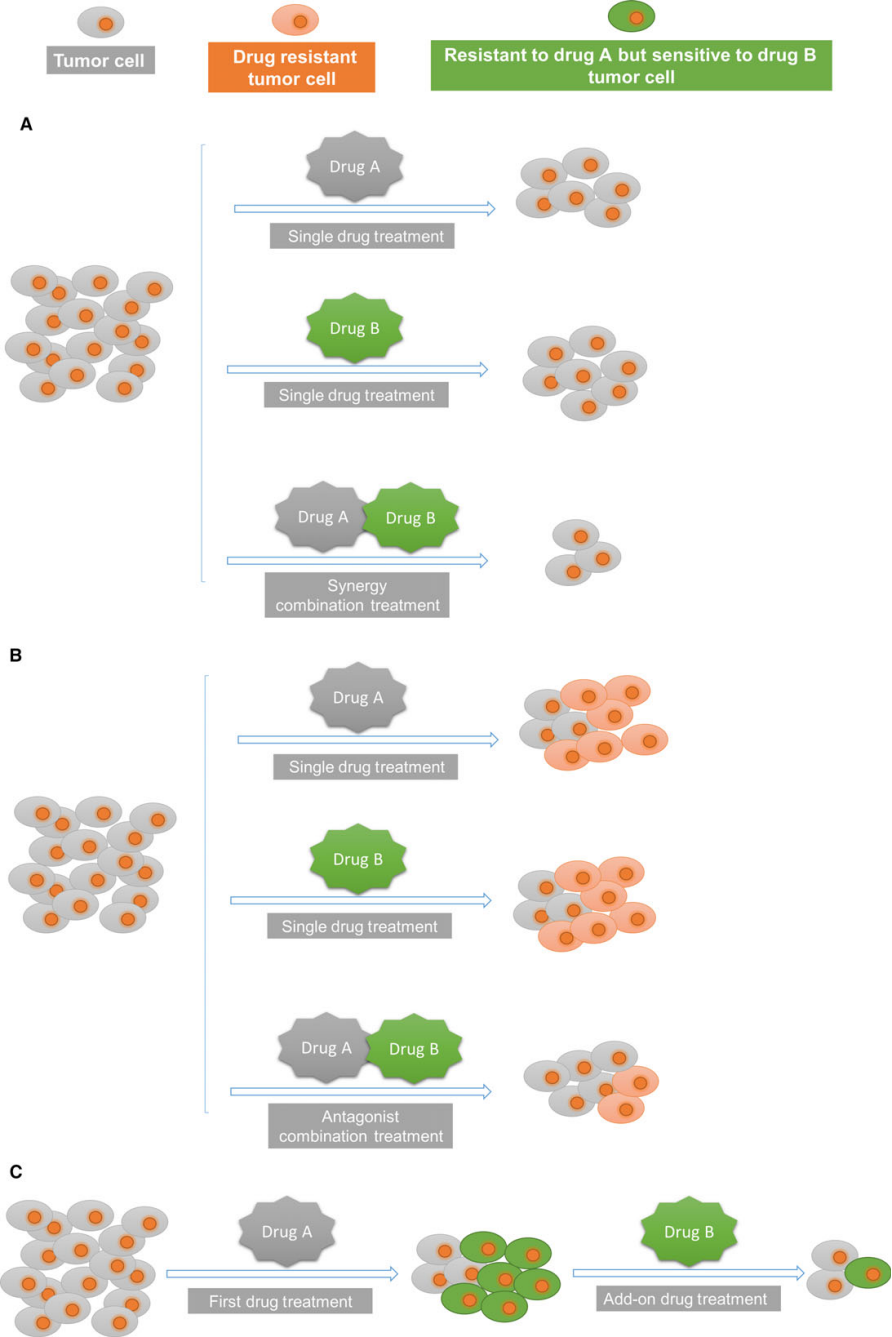
The three drug combination patterns in combination therapeutics. (**A**) The synergy drug combination improves therapeutic effects compared with the single drug treatment. (**B**) During long‐term treatment, drug resistance increases with time. The antagonist drug combination limits the evolution of drug resistance and results in improved therapeutic effects. (**C**) After long‐term drug A treatment, collateral responses increase resistance to drug A and decrease resistance to drug B. Therefore, the addition of drug B to treatment will improve the therapeutic effect.

In the clinic, physicians often face the following pragmatic question: should I use a second drug as an add‐on instead of using more of the first drug? In the case of complex diseases, the answer will often be that an increased amount of the first drug cannot produce the effect that is desired by the physician, but this effect can be achieved with combination treatment 28. Moreover, in some cases, the addition of more of the same drug will induce unacceptable negative influence. For example, when the dosage of the first drug is slightly under the threshold of toxicity, even a small dosage increase may be unacceptable 45. Therefore, combining the first medication with a second drug that produces non‐overlapping toxicities may be beneficial, even when the combined effects on efficacy are only Loewe additive 34, 35, which refers to a model of drug combination and will be introduced in the next paragraph. Ultimately, the ideal design of combination therapeutics must reflect the requirement of the physician that, when drugs are used in combination, an extra benefit is observed that cannot be achieved by using the constituent drugs alone. The extra benefit is mainly derived from the synergistic effects of the combined drugs (Fig. 2A).

The word synergy is derived from the Greek words sunergia, which means ‘cooperation’, and sunergos, which means ‘working together’ 19. Modern pharmacology has embraced this concept to emphasize the idea that a synergistic combination provides greater effects than those predicted by simply adding the effects of individual parts 10. Numerous investigators have attempted to provide a framework to calculate the additive effects among constituents 46, 47, 48. Among these works, the Loewe additivity 49, 50 and Bliss independence 46, 51 models are two of the most useful frameworks of synergy.

The Loewe additivity model assumes that two drugs act through similar mechanisms. Equipotent dose ratios determine the effects of each drug and their combination. According to this model, the combined activity level, *L*_*x*,*y*_, of the two drugs X and Y at the concentrations, *C*_*x*_ and *C*_*y*_, satisfies the equation, $\frac{C-x}{C(L_{x}=L_{x,y})}+\frac{C-y}{C(L_{y}=L_{x,y})}=1$. In this equation, *C*_*x*_ and *C*_*y*_ are the single‐agent concentrations for X and Y, which individually produce an activity of *L*_*x*,*y*_.

The Bliss independence model assumes that two drugs act through independent mechanisms. The combination effect in this model is represented as the union of two probabilistically independent events 46. According to this model, the combined activity, *L*_*x*,*y*_, of two compounds X and Y at the concentrations, *C*_*x*_ and *C*_*y*_, satisfies the equation, *L*_*x*,*y*_ = *L*_*x*_ + *L*_*y*_ − *L*_*x*_ *L*_*y*_. In this equation, *L*_*x*_ and *L*_*y*_ are the single‐agent activity levels at concentrations *C*_*x*_ and *C*_*y*_.

These two models produce different outcome, and only the Loewe additivity model correctly predicts the cases in which the two compounds are actually the same constituent. However, the debate continues over which model performs better in cases involving noisy clinical data and uncertain therapeutic mechanisms 46, 52, 53.

Nonetheless, synergy is not the only reason for drug combinations. Sometimes the combination of antagonistic drugs may also be beneficial (Fig. 2B). In fact, the combination can have two conflicting effects: it may reduce the evolution of drug resistance because it cures the disease faster, thereby limiting the time window available for drug resistant mutations to accumulate, but it may also increase the selective advantage of drug‐resistant mutants 54. If a strong resource competition is present, the latter effect will dominate 54. In fact, under these conditions, antagonistic drug combinations, which are less potent than the sum of the components, have been shown to limit the evolution of drug resistance 55, 56, 57. The advantage of antagonistic combinations over synergistic combinations is the result of a reduced fitness gain of drug‐resistant mutants (Fig. 2B) 55, 56, 57.

In addition to synergistic and antagonistic drug interactions, collateral responses among drugs also play a role in combination therapeutics (Fig. 2C) 58. Collateral response (sensitivity or resistance), also known as cross‐resistance, is the ability of one drug to increase or decrease sensitivity to another drug (Fig. 2C). The study of collateral susceptibility changes started in the early 1950s 59 and has been reported for many different drugs in subsequent years 60, 61, 62. Recently, Munck *et al*. studied the development of resistance against drug combinations by investigating collateral responses to component drugs 58. The authors provided a framework for the rational selection of drug combinations that limit resistance evolution using *Escherichia coli*.

In general, the benefits of combination therapeutics mainly result from synergistic effects. Antagonistic effects and collateral responses may also produce extra benefits by decreasing drug resistance. Therefore, combination therapeutics can be more complicated than simply taking different drugs together. To investigate the rationale of combination therapeutics, preclinical combinational studies should be performed prior to the initiation of clinical trials.

## The development of combination therapeutics in cell lines and animal models

A common strategy to investigate combination therapeutics prior to the initiation of clinical trials is through the use of cell lines and animal models. These models provide important insights into the mechanisms of action and interactions between drugs, which are important in the preparation for clinical trials 63. For example, DiCosimo *et al*. estimated the efficacy of combined ridaforolimus and dalotuzumab for the treatment of breast cancer in human cell lines and mouse models, and the results of those studies led to the initiation of a phase I clinical trial 64. In fact, preclinical studies make up the majority of studies investigating (Fig. 1) and play an essential role in the development of combination therapeutics 65. In general, preclinical studies of combination therapeutics rapidly assess the synergy of combined drugs and explore physical interactions among them. They also evaluate anti‐disease activity, mechanisms of action and additional factors, such as the effects of the drugs on their targets and their pharmacokinetics 66.

However, despite the large number of preclinical combination therapeutics studies, the number of successful clinical trials is limited (Fig. 1). It appears that the success or failure of clinical trials examining combination therapeutics can hardly be predicted on the basis of published preclinical data 67. The lack of concordance between preclinical studies and clinical trials is not surprising. Preclinical studies are limited in experimental design and animal models 63. In experiments that are designed to evaluate the activity of combined drugs, assessments are performed with limited consideration of how the drugs are likely to function in patients 63. Such evaluations can provide insights into the mechanisms responsible for anti‐disease actions and the interactions between the drugs within the parameters of the experiment. However, interpretation of the evaluations and the subsequent design of clinical trials for complex diseases must encompass numerous variables that might exceed the designed parameters of preclinical experiments, such as drug dose, scheduling and route of administration 68.

Even when researchers have thoroughly designed all of the parameters of preclinical experiments for combination therapeutics, the inherent limitations of animal models continue to weaken the correlation between laboratory and clinical outcome. The most important limitation is that the drug targets are host components and are therefore of animal, not human, origin 65. In other words, a drug that works well in an animal model may not necessarily work in humans. After assessing the correlation between cancer animal models and the clinical activity of single cytotoxic drugs, previous studies have suggested that the predictive value of cytotoxic drugs in murine models is limited 69, 70. Limiting experimentation to a few animal models cannot reflect the diversity of complex human diseases and may explain the failure of clinical trials based on preclinical data 67. A detailed understanding of human biology may enable the design of *a priori* combination therapeutics to increase the success rate of clinical trials. A growing number of scientists in the field of therapeutic discovery are attempting to understand biology and disease using computational approaches 71.

## The development of combination therapeutics using computational approaches

Computational approaches, particularly those integrating data analysis and artificial intelligence, may assist in the design of combination therapeutics by generating drug**–**target interaction networks and predicting drug combinations. Computation assistant construction of drug**–**target interaction networks mainly refer to computational algorithms and programs to reveal molecular mechanisms and connections between drugs and targets in a dynamic network 72. These methods can be used to explore the therapeutic mechanisms underlying drug treatment at the molecular level 73. Common methods of this type include knowledge‐ and ligand‐based approaches.

Knowledge‐based approaches are high‐throughput computational methods of artificial intelligence that enable computers to learn from the available knowledge, including drug chemistry and structural information, as well as drug**–**target networks, to predict new knowledge, such as new drug indications, targets and drug**–**target interactions 74. These approaches can be classified into unsupervised and supervised methods 75. The unsupervised methods extract and predict patterns and interactions based on a series of input variables. The commonly used unsupervised methods include clustering, data compression and outlier detection methods such as principal‐component‐based methods 76. For example, Guimera *et al*. studied novel drug**–**drug interactions using a large‐scale unsupervised method that deals with various types of data to accurately predict adverse, synergistic and antagonistic drug interactions 77. Kissa *et al*. examined drug**–**gene associations and assisted in drug repositioning with a fully corpus‐based unsupervised method that utilizes the available knowledge 78. The supervised methods divide the data into training and validation datasets, and they finalize a robust model that can be used to predict the binding probability between drugs and targets 79. The most widely used supervised methods are the Bayesian model 80, the support vector machine 81 and the decision tree 82. These methods can be combined if appropriate. For example, Kim *et al*. predicted drug**–**target interactions by applying two machine learning approaches, a support vector machine and a kernel‐based L1‐norm regularized logistic regression, to evaluate drug**–**drug interaction data 83.

Ligands are small molecules that bind to a site on a target protein to alter the biological functions of the protein. Ligand‐based approaches predict drug**–**target interactions by means of chemical or structural ligand similarities, with the basic assumption that similar ligands tend to bind to similar targets 84. Ligand‐based approaches are highly dependent on the availability of chemical and structural information on targets, from either wet experiments or numerical simulations 85, 86. The similarity ensemble approach 87, the pharmacophore model 88, docking 89 and quantitative structure‐activity relationships 90 are commonly used methods. Based on these types of methods, Yco *et al*. identified sepiapterin reductase as a new therapeutic target for the treatment of human neuroblastoma 91.

Knowledge‐ and ligand‐based approaches are often combined to achieve better results. Afzal *et al*. combined multiple methods to construct drug**–**target predictions and obtained more applicable results 92. These computational approaches accelerate the acquisition of drug**–**target data. The accumulation of these data, once again, promotes novel predictions of drugs targets 93.

In addition to accelerating the accumulation of drug targets, computational approaches directly predict new combination therapeutics. Bansal *et al*. assessed 32 computational approaches and found that the computational prediction of drug combination activity is possible 94. The prediction of new combination therapeutics should include network pharmacology that encompasses systems biology, network analysis, connectivity, redundancy and pleiotropy 95. Gu *et al*. predicted a drug combination in the context of the pathway network of the biological process 96. The computational approach may become an important complement to traditional preclinical studies. It is possible that computational approaches will be used to identify promising combinations that can be tested *in vitro* to further determine the combinations, which would be advantageous compared with random combination screening *in vitro*. This method may even replace *in vitro* screening in cell lines in the future, but *in vivo* efficacy testing would be still required. Large and complex biological data and multiple computational methods are often used in studies focused on the prediction of combination therapeutics 97. With the accumulation of chemical, biological, ‘omics’ data and the advancement of artificial intelligence, computational approaches will acquire a more prominent role in the development of combination therapeutics for complex diseases.

## Conclusions

The use of combination therapeutics is an appropriate approach to address the complexity of human diseases. The advantages of combination therapeutics are mainly due to the ability of combination products to act on multiple targets. Chemical drugs, biological drugs and drugs from natural products are widely used in combination therapeutics. Drug interactions, including synergistic and antagonistic drug interactions, as well as collateral responses, might influence the therapeutic actions of drug combinations. Preclinical studies in cell and animal models have been performed to evaluate the effects of drug combinations. However, subsequent successful clinical trials of drug combinations are limited, potentially because the limited experimental conditions in preclinical studies cannot reflect the diversity of complex diseases. Computational approaches can complement traditional preclinical studies. The use of computational approaches not only facilitates an understanding of drug interactions, but it also promotes the discovery of new combination therapeutics. Computational approaches might help to define the number of candidate combinations for further *in vitro* and *in vivo* investigations. In future, the accumulation of multiple types of human ‘omics’ data, including genomics, transcriptomics, proteomics and metabolome, among others*,* will deepen our understanding of complex diseases and promote the computational discovery of combination therapeutics.

## Conflict of interest

The authors declare that there are no conflicts of interest.
